# Response To Comment on: “A Systematic Review and Meta-Analysis of the Association Between ACTN3 R577X Genotypes and Performance in Endurance Versus Power Athletes and Non-Athletes”

**DOI:** 10.1186/s40798-025-00873-2

**Published:** 2025-06-03

**Authors:** El Mokhtar El Ouali, Benjamin Barthelemy, Juan Del Coso, Anthony C. Hackney, Ismail Laher, Karuppasamy Govindasamy, Abdelhalem Mesfioui, Urs Granacher, Hassane Zouhal

**Affiliations:** 1 Department of Biology, Laboratory of Biology and Health, Ibn Tofail University of Kenitra, Kenitra, Morocco; 2 Sport, Health and Sciences laboratory (M2S). UFR-STAPS, University of Rennes 2-ENS Cachan, Av. Charles Tillon, Movement, Rennes Cedex, 35044 France; 3 Centre for Sport Studies, Rey Juan Carlos University, Fuenlabrada, Spain; 4 University of North Carolina, Chapel Hill, NC USA; 5 Department of Anesthesiology, Pharmacology, and Therapeutics, Faculty of Medicine, University of British Columbia, Vancouver, Canada; 6 Department of Sports, Recreation and Wellness, Symbiosis International (Deemed University), Hyderabad Campus, Modallaguda (V), Nandigama (M), Rangareddy, Telangana 509217 India; 7 Department of Sport and Sport Science, Exercise and Human Movement Science, University of Freiburg, Freiburg, Germany; 8 Institut International des Sciences du Sport (2IS), Irodouer, 35850 France

Dear Editor,

We greatly appreciate Mr. Bottura’s thoughtful commentary letter [1], as his detailed insights provide a unique and valuable perspective on interpreting the association between the *ACTN3* R577X polymorphism and athletic performance. We also appreciate his efforts to clarify specific aspects of our article [2] and to emphasize the importance of a nuanced interpretation in the practical application of genetic data to sports.

## Strength/Power Sports

According to the commentary of Mr. Bottura [1], it is not appropriate to conclude that athletes with the RR genotype have a distinct advantage in strength/power sports. While we appreciate his perspective, we want to strengthen and elaborate our argument that athletes with the RR genotype do have an advantage in strength or power sports. First, in the context of the *ACTN3* R577X (rs1815739) polymorphism, the R allele is considered the wild-type allele, whereas the X allele is regarded as the rare variant (Table 1). Although the frequency of these alleles vary across populations with differing ethnic backgrounds and geographical locations, the RX genotype is, overall, the most common genotype globally [3]. Supporting this, studies utilizing the *Actn3* knockout (KO) mouse model have demonstrated that *ACTN3* R577X influences α-actinin-3 sarcomeric composition in a dose-dependent manner. This evidence we feel suggests that the additive model (RR > RX > XX) is the most appropriate framework for investigating associations between *ACTN3* genotypes and phenotypic traits. Given that the RX genotype is most prevalent, and α-actinin-3 content in skeletal muscle follows a dose-dependent pattern [4], it is reasonable to consider the RR genotype as conferring a potential advantage, as it provides a potential differential function over the most prevalent RX genotype. Second, our meta-analysis showed that the RR genotype was overrepresented (see Fig. 6 of the original article [2]) and the XX genotype was underrepresented (see Fig. 8 of the original article [2]) among strength/power athletes in comparison to controls. This observation aligns with the hypothesis that the RR genotype provides a potential advantage in such sports, or alternatively, that XX athletes may be at a disadvantage. Furthermore, the scientific evidence supporting the view that the RR genotype confers a distinct advantage in strength/power sports, and that XX athletes lack this advantage, is both reasonable and valuable in our opinion [5, 6]. This perspective emphasizes a relative, rather than absolute, effect of *ACTN3* genotypes on power-based sports and can help sports practitioners better understand the role of the *ACTN3* gene in athletic performance. That said, we recognize that incorporating demographic data on the global distribution of the *ACTN3* R577X genotypes in our meta-analysis [2] would have provided a broader and more comprehensive perspective. Including such data could have enriched our study findings and allowed a deeper context to understand the varying associations between *ACTN3* genotypes and power-based performance across various ethnic groups worldwide.

**Table 1:** *ACTN3* R577X (rs1815739) allele frequencies and genotype distributions according to geographic regions using data from the 1000 genomes project [3]

Region	*R* allele	X allele	Most common allele	RR	RX	XX
**Global**	0.60	0.40	R	0.39	0.43	0.18
**Africa**	0.89	0.11	C	0.78	0.22	0.00
**America**	0.43	0.57	T	0.16	0.53	0.31
**East Asia**	0.56	0.44	C	0.31	0.50	0.19
**Europe**	0.57	0.43	C	0.31	0.51	0.18
**South Asia**	0.41	0.59	T	0.16	0.51	0.33

## Endurance Sports

Mr. Bottura [1] also suggests that expression of α-actinin-3, associated with RR and RX genotypes, may be beneficial for endurance sports, in contrast to the XX genotype that may be considered detrimental. This view reflects that the R allele (or the RR genotype) may be equally beneficial for both power-based and endurance-based sports. We have a different view on the potential associations of the *ACTN3* R577X polymorphism with endurance sports as we do not consider the RR genotype as being beneficial for aerobic sports. We have this perspective for several reasons. First, we did not contrast endurance athletes with non-athletic controls in our meta-analysis. Therefore, it is unfeasible to ascertain with our study if the RR genotype is distinct in endurance athletes vs. non-athletic controls. However, interestingly, we found that the RR genotype was overrepresented in power athletes vs. endurance athletes (see Fig. 10 of the original article [2]) which contradicts the hypothesis that the RR genotype may be equally beneficial for both power-based and endurance-based sports. Additionally, previous systematic reviews have compared endurance athletes with non-athletic controls, and they have concluded that the influence of the *ACTN3* R577X polymorphism is small [7]. In that case, it seems that the XX genotype may offer little advantage for endurance performance. Along these lines, the *Actn3* KO mouse model has reflected that there is a shift towards a more efficient aerobic muscle metabolism coupled with an improved recovery from fatigue in *Actn3* KO mice, suggesting a potential advantage of the X allele or the XX genotype for endurance sports performance [5]. In our study, the XX genotype was also more frequent in the population of endurance vs. power athletes (see Fig. 13 of the original article [2]) which indirectly supports this view. Collectively, we consider that the influence of the *ACTN3* R577X polymorphism on phenotypes associated with endurance sports to be small while the potentially beneficial genotype would be XX rather than RR.

We also value the examples of elite athletes cited by Mr. Bottura [1] to illustrate the possible associations between *ACTN3* genotypes and athletic performance. However, we wish to emphasize that we do not have specific genetic data on these athletes. Thus, directly linking their exceptional performance to the *ACTN3* genotype should be approached with caution. Moreover, while genetics plays an important role in physical abilities, the performance of these elite athletes likely results from the interaction of several factors, including environment, physical and psychological preparation, and socio-cultural and economic factors. These elements, combined with potential genetic predispositions, shape the remarkable performances of the athletes mentioned. We encourage future research to integrate these multiple dimensions to better understand the interaction between genetics and environment in elite sports performance.

We would like to express our sincere gratitude to Mr. Bottura [1] for his insightful remarks and his in-depth analysis of our article. His comments provide valuable perspectives on the application of genetic data in sports, particularly regarding the interpretation of *ACTN3* genotypes in specific performance contexts. We appreciate the opportunity to engage in a constructive scientific dialogue on this complex issue and hope that this discussion will contribute to enriching the understanding and importance of further scientific contributions to genetics in sports.

## Data Availability

Not applicable.

## Abbreviations

ACTN3: Alpha-actinin-3
ACTN3 R577X: ACTN3 gene polymorphism
R allele: allele of the ACTN3 gene polymorphism associated with ACTN3 production
RR: homozygous ACTN3 genotype associated with ACTN3 production
RX: heterozygous ACTN3 genotype
XX: homozygous ACTN3 genotype that does not produce ACTN3
